# Statement of Retraction: effect of galectin-3 in the pathogenesis of arteriovenous fistula stenosis formation

**DOI:** 10.1080/0886022X.2026.2740592

**Published:** 2026-10-05

We, the Editors and Publisher of Renal Failure, have retracted the following article:

Ruan, L., Yao, X., Li, W., Zhang, L., Yang, H., Sun, J., & Li, A. (2021). Effect of galectin-3 in the pathogenesis of arteriovenous fistula stenosis formation. *Renal Failure*, 43(1), 566–576. https://doi.org/10.1080/0886022X.2021.1902822

Since publication, concerns were identified by a third-party regarding the integrity of Figure 2, 4B, and 5 A. Further investigation by the publisher and journal has confirmed the integrity concerns. When approached for an explanation, the authors provided the relevant images; however, concerns remain.

As verifying the validity of published work is core to the integrity of the scholarly record, we are therefore retracting the article. The corresponding author has been informed.

We have been informed in our decision-making by our editorial policies and the COPE guidelines.

The retracted article will remain online to maintain the scholarly record, but it will be digitally watermarked on each page as ‘Retracted’.

